# Distraction Modulates Self-Referential Effects in the Processing of Monetary and Social Rewards

**DOI:** 10.3389/fpsyg.2018.02723

**Published:** 2019-01-09

**Authors:** Jia Zhu, Youlong Zhan

**Affiliations:** ^1^Department of Psychology, Hunan Normal University, Changsha, China; ^2^Department of Psychology, Hunan Agricultural University, Changsha, China; ^3^Department of Psychology, Hunan University of Science and Technology, Xiangtan, China

**Keywords:** self-relevance, attention, social reward, monetary reward, ownership

## Abstract

A reward that is personally relevant tends to induce stronger pursuit motivation than a reward that is linked to other people. However, the role of attention in eliciting this “self-referential reward effect” remains unclear. In our two studies, we evaluated the significance of attention in self-referential reward processing utilizing an ownership paradigm, which required participants to complete a visual search task to win either monetary rewards (in Study 1) or social rewards (in Study 2) for themselves or for an acquaintance. Access to attentional resources was manipulated by sometimes including a distracting stimulus among the presented stimuli. The results of Study 1 revealed that a significant self-referential reward effect emerged under undistracted attentional conditions and was associated with improved task performance when self-owned monetary rewards were available. However, distracted attention impaired this self-referential reward effect. Moreover, distracted attention was also observed in the self-referential social reward processing in Study 2. These results suggested that distracted attention can impair the pursuit advantage for self-relevant rewards; self-referential processing is strongly dependent on attentional resources.

## Introduction

The influence of the self on attentional processes has been recognized by psychologists. It has been proposed that humans are equipped with a mechanism that enables self-relevant information to be attended to rapidly and reliably (Gray et al., 2004; Sui et al., 2006; Turk et al., 2011a). Numerous studies have reported that when a stimulus is cued as being relevant to one’s self, event-related potentials (ERPs) suggest a rapid increase in both visuospatial and executive attention to the stimulus (Herbert et al., 2011; Turk et al., 2011b; Fan et al., 2013; Northoff, 2016; Sui and Gu, 2017). The tendency for self-cues to capture attention is clearly advantageous, as information that is coupled with the self is likely to be of greater personal importance than material linked with other people. Reflecting this potential importance, a reward associated with oneself elicits a robust motivating advantage compared to a reward linked to another person (Krigolson et al., 2013; Sui and Humphreys, 2015; Zhan et al., 2016). Thus, the question of interest in the present study is whether the increased motivation to earn self-relevant rewards depends on the attentional resources devoted to self-cues. A number of studies have reported self-referential effects in reward processing and suggested that self- and reward-related processing either interacted or were independent in various external contexts (Northoff and Hayes, 2011). For instance, compared to other-relevant reward stimuli, self-relevant reward stimuli can be perceived faster during a series of perceptual matching tasks (Sui et al., 2015a,b), result in faster learning of reward rules in a social gambling task (Kwak et al., 2014), elicit larger P2 and P3, which indicate stronger respective attentional salience and motivational importance (Martín et al., 2016; Zhan et al., 2017), and evoke stronger neural activations in the reward region of the brain (Hassall et al., 2016). Therefore, these findings are consistent with the general notion that self-relevant items often have higher intrinsic value within individuals’ subjective value systems compared to stimuli related to other people (Northoff, 2016). However, it is somewhat difficult to apply this to the nonevaluative self-referential reward effect described above, as participants were not required to relate the incoming rewards to the self. This theoretical gap could be bridged by consideration of the importance of attention in processing. Given the attention capture known to follow the perception of self-cues, this may enhance resource-intensive processing in self-referential processing contexts, such as owning objects and making outcome choices. This should elicit stronger pursuit motivation for self-relevant rewards relative to other-relevant rewards and improve subsequent task performance.

Supporting this reasoning, our study argues that self-referential reward effect can be described as a classical “endowment effect” (Hassall et al., 2016) that is dependent on the accessibility of attentional resources (van den Bos et al., 2010). Elaboration of incoming stimuli tends to be a process requiring additional attentional resources. For instance, previous studies have reported that distraction can influence the processing of emotional stimuli, as well as emotional regulation strategies (Paul et al., 2013; Li and Yuan, 2018). Moreover, studies on self-referential memory have observed that separating attention dramatically lowered recognition (Gardiner and Richardson-Klavehn, 2000; Gardiner et al., 2001; Turk et al., 2013). Such findings indicated that elaborative self-referential memory representations depended, to a greater extent, on the application of attentional resources at encoding compared to representations associated with other people. Additionally, Hickey and his colleagues have also demonstrated that distracted attention impairs the motivating effects of stimuli associated with rewards (Hickey et al., 2006, 2009, 2010). Specifically, participants’ performance decreased when target stimuli were accompanied by distracting stimuli during a visual search task. These findings suggested that available attentional resources can impact the self-referential effect during the processing of rewards. Hence, we speculated that attentional allocation may be the mechanism that enables the existence of the self-referential reward effect, in which self-owned rewards evoke stronger pursuit motivation than other-owned rewards in full-attention conditions. However, distracted attention could impair this effect due to limited attentional resources.

To further confirm this speculation, the present study included two studies (using monetary and social rewards) to identify whether inadequate resources have a selectively deleterious role on the self-referential reward effect. More specifically, we predicted that self-relevant monetary rewards might require resource-intensive processing and would therefore be affected by distracted-attention (DA) manipulations (Study 1). Consequently, we expected that only monetarily rewarded visual searches would decrease under DA conditions and not unrewarded visual searches. Furthermore, we examined whether there was a similar impairing effect of distracted attention on the self-referential social reward effect in Study 2.

## Study 1

### Sample Population and Design

In this study, 89 right-handed college students (42 males and 47 females aged 17–23 years) were included. All participants had normal or corrected-to-normal vision and no known neurological impairments. Additionally, all participants were volunteers from Hunan Normal University and received academic credit in their undergraduate psychology courses for participation. We obtained specific informed consent from each participant for publishing information or images that could potentially reveal their identities in an online open-access publication. A 2 (referential cues: self-referential cue, other-referential cue) × 3 (reward cues: high monetary reward cue, low monetary reward cue, non-reward cue) × 2 (attentional condition: distracting stimulus, no distracting stimulus) within-subjects design was employed. All experimental procedures were conducted in accordance with the Declaration of Helsinki and approved by the Ethics Committee of Hunan Normal University. After fully understanding the study, each participant signed an informed consent form.

### Stimuli and Tasks

#### Self-Association Task

Prior to the experiment, each participant was provided a name of an acquaintance with whom they were unfamiliar but greeted upon meeting. Throughout the coupling task, the red circle and triangle geometric shapes were, respectively assigned to labels representing the participant and the acquaintance in two sessions. For instance, participants were informed that “the triangle is your acquaintance” and “the circle represents yourself” (Sui et al., 2012). Among all participants, the orders of the shape-labeled pairs presented were randomized, and the associations between shape and label were counterbalanced. Following the associative directions, participants underwent a shape-label matched pair. A shape (covering 3.5° × 3.5° visual angle) was presented above a white central fixation cross (0.8° × 0.8° visual angle). Of the two labels, one (the name of the participant and their acquaintance) (covering 1.76°/2.52° × 1.76° of visual angle) was shown beneath the fixation cross. Participants were assigned the tasks of determining whether the shape-label pair was equivalent as initially shown or if the shape and label were altered. Participants were informed that they could only go on to the next stage if they responded correctly in all of the trials; otherwise, the cycle was repeated.

#### Visual Search Task

**FIGURE 1:**
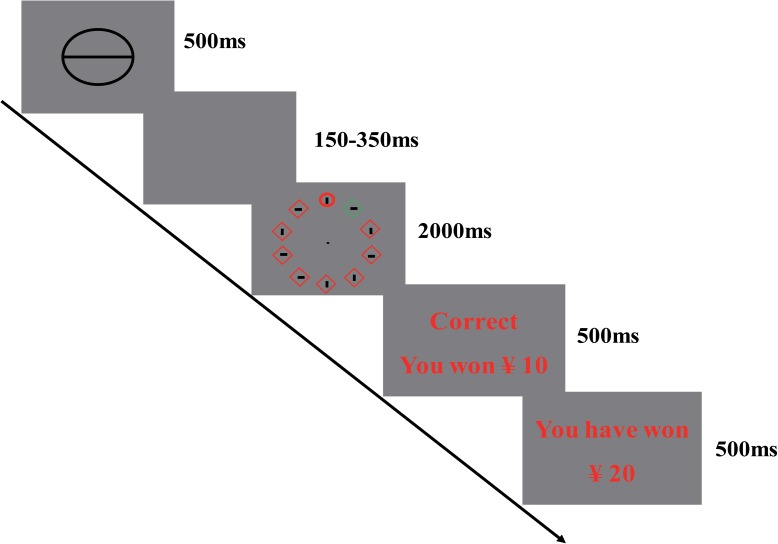
Sequence of events in the visual search task in Study 1.

The visual search arrays included 10 object outlines (line thickness of 0.3° visual angle), each appearing equidistant (9.1°) from a central fixation point and from the other. Objects were either diamonds (4.2° × 4.2°) or circles (3.4° diameter), while each display contained only a singular uniquely shaped item. The unique item was either a diamond (while all other stimuli were circles) or a circle (while all other stimuli were diamonds). Among 80% of the trials, one of the homogenously shaped nontarget items were of unique color, such as red (with all other objects green) or vice versa. For each trial, distractor and target colors were determined randomly. A gray line, which was randomly oriented vertically or horizontally, was contained for each object (0.3° × 1.5°). The sequence of events on the visual search task is presented in Figure 1. The task began with a fixation cross presented for 500ms, followed by a cue for 1000ms. Each potential reward size differed on three levels, as stated by the number of parallel lines in the cue. Absence of a reward was identified by an empty white circle, while a low reward (a value of 10 Yuan) was identified by a white circle with a horizontal line and a high reward (a value of 20 Yuan) was identified by a white circle with two horizontal lines. After a random interval of 600–1000 ms, the visual search task was presented for 4000ms. Feedback stimuli were shown for 500 ms following a variable target-feedback interval of 800–1000 ms, based on the reward magnitude for the present trial and the participant’s response. In the case of no-reward trials, a “√” or “×” was presented as a feedback stimulus based on whether the button pressed was correct. In monetary reward trials where participants provided quick correct responses, monetary rewards of 10 or 20 Chinese Yuan banknotes were given as feedback stimuli. Finally, the cumulative points of the participant and their acquaintances were presented for 500 ms. During that task, participants were informed of their performance by viewing the cumulative points for reward feedback stimuli.

### Procedure

The full experiment includes the self-association task and the visual search task. Before the experiment, all participants completed some exercises to become familiarized with the procedures. During the self-association task, participants formed two special pairs between shapes (the red circle and triangle geometric shapes) and labels (the self and acquaintance). They were then given a two-minute break, after which the participants were asked to complete the visual search task for monetary reward. Finally, participants were asked to complete a 7-point subjective rating regarding motivation for the three types of reward cues; for example, answering “how much do you desire to correctly respond to the cue stimuli?” with ratings ranging from 1 (no desire at all) to 7 (strongly desire). Stimuli presentation and the recording of RTs and ACC were accomplished using E-Prime 2.0 software (Psychology Software Tools, Pittsburgh, PA). The full experiment took about 25 min.

### Data Analysis

Using repeated-measures of analysis of variance (ANOVA), RTs and accuracy were separately analyzed based on within-subjects factors, which were referential cues (self-referential and other-referential), reward cues (high monetary reward, low monetary reward, and no reward), and attentional condition (distracting stimulus, no distracting stimulus). All data analysis was performed using Statistical Package for the Social Sciences (SPSS) Version 20.0 (IBM Inc., New York, United States). Partial *η^2^* was shown as an effect size estimate. For all significant interactions, we conducted *post hoc* pairwise comparisons with Bonferroni adjustments.

### Results

The analyses of subjective rating scores revealed that the 20 Yuan ($2.88) was more strongly desired than the 10 Yuan RMB ($1.44), *t*_1,87)_ = 6.13, *p* < 0.001. Also, the 10 Yuan RMB was more strongly desired than a same-sized blank paper, *t*_(1,87)_= 13.56, *p* < 0.001. These results suggest that monetary reward was operationalized successfully.

**FIGURE 2:**
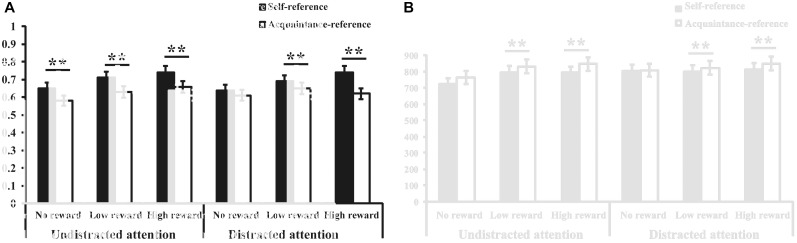
Mean accuracy **(A)** and reaction time **(B)** under all conditions in Study 1. Asterisks represent the significant level, ^∗^*p* < 0.05 and ^∗∗^*p* < 0.01.

Table 1 presents the mean accuracy rates and RTs. The accuracy rates reveal a main effect for the reward cues (*F*_(2,176)_= 27.05, *p* < 0.001, $\eta_{p}^{2}$ = 0.24), with significantly higher average accuracy rates in the monetary reward condition compared to the no-reward condition; however, there were no significant differences between the high and low monetary reward conditions. Additionally, a main effect of referential cues (*F*_(1,88)_= 51.60, *p* < 0.001, $\eta_{p}^{2}$ = 0.37) suggested a significantly higher average accuracy rate for the self-referential cues than for the acquaintance-referential cues. There was also a significant three-way interaction between reward cues, referential cues, and attentional condition, *F*_(2,176)_= 4.32, *p* < 0.05, $\eta_{p}^{2}$ = 0.11. Further simple effect analysis revealed that in the undistracted condition, participants demonstrated higher average accuracy rates for the self-referential cues than for the other-referential cues under three kinds of rewards conditions (*Fs*_(1,88)_= 15.60–18.75, *ps* < 0.001). However, in the distracted conditions, such self-referential reward effect was reduced under both high and low monetary rewards conditions (*F*_(1,88)_= 10.08, *p* < 0.001, *F*_(1,88)_= 15.60, *p* < 0.001) rather than in the no-rewards conditions, *F*_(1,88)_= 2.79, *p* > 0.05 (Figure 2A).

**Table 1:** Mean and SD of accuracy rate (%) and RTs (ms) in study 1.

		Accuracy rate		RTs	
		
		Self-reference	Acquaintance-reference	Self-reference	Acquaintance-reference
Undistracted attention	No reward	0.65 ± 0.18	0.58 ± 0.22	722.55 ± 171.70	764.02 ± 202.29
	Low reward	0.71 ± 0.20	0.63 ± 0.21	797.38 ± 134.28	832.57 ± 168.45
	High reward	0.74 ± 0.19	0.66 ± 0.19	793.29 ± 124.34	848.28 ± 160.59
Distracted attention	No reward	0.64 ± 0.19	0.61 ± 0.17	804.61 ± 128.70	807.02 ± 159.42
	Low reward	0.74 ± 0.14	0.65 ± 0.15	798.28 ± 134.45	823.84 ± 158.15
	High reward	0.74 ± 0.17	0.62 ± 0.17	811.61 ± 147.88	850.72 ± 126.60


Findings from the RTs show a main effect of reward cue (*F*_(2,176)_ = 18.74, *p* < 0.001, $\eta_{p}^{2}$ = 0.18), presenting an associated increase in RTs in the monetary reward condition compared to no-reward condition, as well as faster RTs for high monetary rewards than for low monetary rewards. Additionally, a main effect of referential cue was observed (*F*_(1,88)_= 8.60, *p* < 0.01, $\eta_{\rho }^{2}$= 0.09), suggesting significantly faster RTs for the self-referential cues than the acquaintance-referential cues. The main effect of attentional condition was significant (*F*_(1,88)_= 24.97, *p* < 0.001, $\eta_{p}^{2}$ = 0.22), suggesting significantly faster RTs for the target stimuli in the undistracted conditions than in the distracted conditions. In addition, we observed a significant interaction between reward cue and referential cue (*F*_(2,176)_= 10.09, *p* < 0.001, $\eta_{p}^{2}$ = 0.10). Further simple effect analysis revealed that in the high and low monetary reward conditions, the participants demonstrated faster RTs for the self-referential cues than for the acquaintance-referential cues (*F*_(1,88)_= 27.12, *p* < 0.001, *F*_(1,88)_= 7.39, *p* < 0.01). However, such differences in RTs were not observed in the no-reward condition (*F*_(1,88)_= 1.87, *p* > 0.05). Furthermore, we found an associated interaction between referential cue and attentional condition (*F*_(2,176)_ = 15.56, *p* < 0.01, $\eta_{p}^{2}$ = 0.11). Additional simple effect analysis revealed that the self-referential reward effect was only observed in the undistracted conditions (*F*_(1,88)_= 14.74, *p* < 0.001) and not in the distracted conditions (*F*_(1,88)_= 1.72, *p* > 0.05, Figure 2B).

### Discussion

The findings from Study 1 indeed show an obvious self-referential reward effect, suggesting that participants’ performances are better (e.g., higher accuracy rate and faster RTs) when earning self-owned monetary rewards as opposed to acquaintance-owned monetary rewards. Importantly, this self-referential reward effect was impaired under distracted attentional conditions. This was observed when self-referential cues, rather than acquaintance-referential cues, were associated with reduced performance when distracting stimuli were present. The pattern of responses observed during the full-attention condition was similar to findings from van den Bos et al. (2010). Specifically, they found that ownership effects are observed during recognition along with recollective experience. Additionally, the authors suggested that the advantage for self-relevant items would diminish when participants were completing a task with divided attention at the time of encoding. Turk et al. (2013) reported that while a self-referential effect in memory was observed under full-attention conditions, the memory benefit was lost during divided attention at the time of encoding. In the present study, self-owned rewards evoked stronger pursuit motivations and were associated with better performance than other-owned rewards in the undistracted conditions. However, the self-referential reward effects were impaired in the distracted conditions. According to literature on the general attentional requirements of elaborative encoding, as well as ownership studies on the patterns of brain activation (Cunningham et al., 2008; Huang et al., 2009; Krigolson et al., 2013), the present findings revealed that ownership effects occur solely when adequate attentional resources are available. However, other studies have reported the similarities and differences between monetary and social reward processing. For example, compared to monetary rewards, participants were given greater cognitive efforts for social rewards (Rademacher et al., 2014), and autistic children showed weaker attentional bias and lower performance for social rewards (Stavropoulos and Carver, 2014; Neuhaus et al., 2015). Recently, Wang et al. (2017) suggested that social and monetary rewards effectively improved performance for all age groups (children, adolescents, and adults) as reward size increased in the selection reaction time task, and social rewards showed resilient subjective incentive power compared to monetary rewards among children and adolescents. In sum, these studies indicated that monetary and social rewards have not only similar enhancement effects for cognitive processes, but also some considerable differences in intensity. However, it remains unclear whether the impairing effect of distraction was observed in the processing of social rewards.

## Study 2

### Participants and Design

In total, 108 right-handed college-aged participants (58 males and 50 females aged 16–24 years) were included in the study. All of the participants’ vision was either normal or corrected-to-normal, and the subjects had no known neurological impairments. Additionally, all participants were volunteers who received academic credit in their undergraduate psychology courses at Hunan Normal University in exchange for participating. A 2 (referential cues: self-referential, other-referential) × 3 (reward cues: high social reward, low social reward, non-reward) × 2 (attentional condition: distracting stimulus, no distracting stimulus) within-subjects design was employed. We expected that when comparing undistracted attentional conditions, the task performance in the presence of both self-referential and acquaintance-referential cues would be reduced in the distracted attentional conditions. All experimental procedures were conducted in accordance with the Declaration of Helsinki and approved by the Ethics Committee of Hunan Normal University. After fully understanding the study, each participant signed an informed consent form.

### Stimuli and Procedure

The same experimental methods from Study 1 were used in Study 2, and the SID task was adopted to examine how self-reference affected the processing of social rewards given different attentional resources. The social rewards were 10 or 20 cartoon smiley faces (low and high social rewards, respectively), and the no-reward stimulus was a neutral cartoon face (Rademacher et al., 2014; Wang et al., 2017). Hence, the only difference from Study 1 was the reward cues.

### Results

The analyses of the subjective rating scores revealed that the 20 cartoon smiley faces were more strongly desired than the 10 cartoon smiley faces (*t*_(1,106)_ = 13.11, *p* < 0.001), and the 10 cartoon smiley faces were more strongly desired than the neutral faces (*t*_(1,106)_= 14.82, *p* < 0.001). These results suggest that the social reward was operationalized successfully.

**FIGURE 3:**
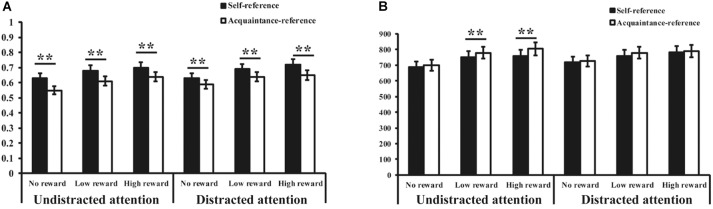
Mean accuracy **(A)** and reaction time **(B)** under all conditions in Study 2. Asterisks represent the significant level, ^∗∗^*p* < 0.01.

Table 2 presents the RTs and mean accuracy rates. The accuracy rates show a main effect of reward cue (*F*_(2,214)_= 22.70, *p* < 0.001, $\eta_{p}^{2}$ = 0.18), presenting significantly higher average accuracy rates for the social rewards compared to no reward, as well as significantly higher average accuracy rates for high social rewards than low social rewards. Additionally, a main effect of referential cue (*F*_(1,107)_= 36.57, *p* < 0.001, $\eta_{\rho }^{2}$= 0.26) suggested a significantly higher average accuracy rate for the self-referential cues than for the acquaintance-referential cues. There were no other significant main effects or interaction effects (each *p* > 0.13; Figure 3A).

**Table 2:** Mean and SD of accuracy rate (%) and RTs (ms) in study 2.

		Accuracy rate		RTs	
		
		Self-reference	Acquaintance-reference	Self-reference	Acquaintance-reference
Undistracted attention	No reward	0.63 ± 0.21	0.55 ± 0.19	687.30 ± 190.67	700.23 ± 197.34
	Low reward	0.68 ± 0.21	0.61 ± 0.18	750.04 ± 175.06	779.24 ± 191.06
	High reward	0.70 ± 0.20	0.64 ± 0.21	758.50 ± 179.11	803.67 ± 185.32
Distracted attention	No reward	0.63 ± 0.18	0.59 ± 0.18	718.24 ± 202.03	725.51 ± 206.41
	Low reward	0.69 ± 0.16	0.64 ± 0.18	759.24 ± 175.07	779.12 ± 196.39
	High reward	0.72 ± 0.15	0.65 ± 0.17	780.69 ± 172.47	789.69 ± 190.45


The results for the RTs showed a main effect of reward cue (*F*_(2,214)_ = 21.23, *p* < 0.001, $\eta_{p}^{2}$ = 0.17), presenting significantly increased RTs for the social rewards compared to no reward and faster RTs for high social rewards than for low social rewards. Additionally, a main effect of referential cue was observed (*F*_(1,107)_= 8.36, *p* < 0.01, $\eta_{p}^{2}$ = 0.10), suggesting associated faster RTs for the self-referential cues compared to the acquaintance-referential cues. A main effect of attentional condition was also observed (*F*_(1,107)_= 4.20, *p* < 0.05, $\eta_{p}^{2}$ = 0.09), revealing significantly faster RTs in the undistracted conditions than in the distracted conditions. Additionally, we observed a significant three-way interaction between reward cue, referential cue, and attentional condition (*F*_(2,214)_= 6.72, *p* < 0.01, $\eta_{p}^{2}$ = 0.06). Further simple effect analysis revealed that in the undistracted conditions, participants demonstrated faster RTs with the self-referential cues than with the other-referential cues under the low and high social reward conditions (*F*_(1,107)_= 6.56, *p* < 0.01; *F*_(1,107)_= 16.36, *p* < 0.001). However, a self-referential reward effect was not observed in the no-reward conditions. Moreover, in the distracted conditions, self-referential reward effects were not observed for any of the reward conditions (*Fs*_(1,107)_= 0.33–0.58, *ps >* 0.05; Figure 3B).

### Discussion

As observed in the first study, the findings from Study 2 further demonstrated that the self-referential social reward effect also was impaired under the distracted conditions. This evidence showed that self-reference could also promote the pursuit of motivation for social reward, but only under full-attentional conditions. However, previous research has shown that individuals demonstrate different behavioral and neural responses while pursuing monetary and social rewards (Stavropoulos and Carver, 2014; Neuhaus et al., 2015). For example, Rademacher et al. (2014) found that participants applied greater cognitive efforts toward earning monetary reward cues compared to social reward cues. Recently, Wang et al. (2017) revealed that both social and monetary rewards successfully improved responding in all age groups (children, adolescents, and adults) as reward size increased in the selection reaction time task, and social rewards showed resilient subjective incentive power compared to monetary rewards among children and adolescents. As seen in Study 1 with monetary rewards, Study 2 also found an obvious self-referential reward effect in the pursuit of social rewards, though divided attention impaired this advantage. However, there were some differences from Study 1. Study 1 found that the impairing effect of divided attention was only observed in the no-reward conditions and not in the low or high reward conditions. Nevertheless, in Study 2, this impairment was observed in all reward conditions. These findings revealed that the impairing effect of distracted attention on self-referential monetary reward effect was modulated by reward value rather than self-referential social reward effect.

## General Discussion

In the present study, we explored the extent to which distracted attention during monetary and social reward pursuit impacts the reward advantage usually associated with self-owned items (Turk et al., 2011b; Krigolson et al., 2013). It was found that under undistracted conditions, an “ownership effect” (i.e., better performance for self-owned than for acquaintance-owned rewards) emerged in the low and high monetary conditions, whereas no effect of ownership was observed in the no-reward conditions. This pattern of responses in the undistracted attention condition replicated the behavioral findings from Krigolson et al. (2013), which reported that ownership effects were observed in a simple gambling task during which participants could “win” or “lose” prizes for themselves or for someone else. Moreover, this self-referential reward effect was observed in the pursuit of social rewards.

Given the effortful nature of elaborative encoding, it was expected that the advantage of self-referenced (i.e., self-owned) rewards would be diminished when participants were completing a distracted-attention task in pursuit of rewards. This pattern was found, with no ownership effect emerging under conditions of undistracted attention. It seems plausible to conclude from this pattern that attentional resources are required when engaging in elaborative reward processing for self-owned objects, which is not possible under conditions of serious resource depletion. This pattern of reward performance augments the evidence that self-referential reward processing, relative to the processing of objects associated with other people, triggers rich, elaborative reward processing (Sui et al., 2015a,b), which is attentionally demanding. To our knowledge, the current study provides the first evidence that the self-reference effect in reward processing is underpinned by differences in attentional processing during the pursuit of rewards.

Interestingly, pursuit of acquaintance-owned rewards was unaffected by distracted attention, suggesting that relatively little elaboration of other-owned rewards takes place, even under undistracted attentional conditions. It is possible that with more power, a small effect of distracted attention would also have been observed in the pursuit of acquaintance-owned rewards, as some elaboration would be expected to support reward processing in this condition. However, the interaction between self-reference and attention was expected, because distracted attention should have a larger effect on self-referential reward processing, reducing the elaboration with which it is distinguished. Importantly, the value of the reward could modulate the influences of self-reference and attention on the pursuit of monetary rewards rather than social rewards. This impairing effect of distracted attention on self-referential reward effect was weak or nonexistent with greater value of monetary rewards. However, this modulation of reward value was not observed in the pursuit of social rewards. We speculate that college students might not be as sensitive to social rewards as they are to monetary rewards. College students are gradually integrating into society and pay increasing attention to money due to their increasing economic independence. Therefore, the value of monetary rewards has an advantage for motivating behavior, compared to social rewards such as cartoon smiles, among college students (Wang et al., 2017). Moreover, this could also be because the incentive value of the social reward was reduced by repeated presentation (Demurie et al., 2012).

These novel findings suggesting that attention is crucial for the self-referential reward effects casts new light on the potential links between the ways in which the self influences human cognition. In particular, the link between the dependence on attention for self-reference effects and the well-known attentional-capturing effect of self-cues during reward processing is an interesting theoretical angle. The two effects could be causally related, as the attention recruited by self-cues could be the mechanism by which resource-dependent elaborative processing and proper motivation were evoked. Cues relating to other people, which do not attract the same degree of attention, could fail to benefit from these reward-enhancement processes; hence, distracted attention has little effect on pursuit motivation by other-relevant rewards. Moreover, this modulation of attention could be explained by the parallel relationship between self and reward, as the self-referential reward effect was indeed affected by the availability of attentional resources. However, the present study did not assess reward seeking motivation. According to reinforcement sensitivity theory, individuals show differences in subjective sensitivity and behavioral approaches toward earning the same reward (Jackson, 2003). Thus, future studies on self-referential reward effects should consider individual differences in reward sensitivity.

## Conclusion

The current study indicated that the processes underlying the monetary and social rewards of elaborative encoding and pursuit in response to self-cues are attentionally demanding. While an ownership effect is elicited under undistracted attentional conditions, this self-referential reward effect was impaired when attention was divided by distracting stimuli. Moreover, the impairment caused by distracted attention was weaker or nonexistent along when the value of monetary rewards, rather than social rewards, increased. The present study offers clear evidence that the reward enhancement associated with self-referential reward processing is dependent on the availability of attentional resources.

## Author Contributions

JZ and YZ designed the experiments and wrote the manuscript. JZ recruited participants and collected the data. YZ performed the data analyses.

## Conflict of Interest Statement

The authors declare that the research was conducted in the absence of any commercial or financial relationships that could be construed as a potential conflict of interest.
